# Participatory Design of a Medication Module in an Electronic Medical Record for Paediatric Palliative Care: A Think-Aloud Approach with Nurses and Physicians

**DOI:** 10.3390/children9010082

**Published:** 2022-01-06

**Authors:** Sven Kernebeck, Chantal Jux, Theresa Sophie Busse, Dorothee Meyer, Larissa Alice Dreier, Daniel Zenz, Boris Zernikow, Jan Peter Ehlers

**Affiliations:** 1 Faculty of Health, Witten/Herdecke University, 58448 Witten, Germany; chantal.jux@uni-wh.de (C.J.); theresa.busse@uni-wh.de (T.S.B.); jan.ehlers@uni-wh.de (J.P.E.); 2 PedScience Research Institute, 45711 Datteln, Germany; d.meyer@pedscience.de (D.M.); l.dreier@pedscience.de (L.A.D.); b.zernikow@kinderklinik-datteln.de (B.Z.); 3 Department of Children’s Pain Therapy and Pediatric Palliative Care, Faculty of Health, School of Medicine, Witten/Herdecke University, 58448 Witten, Germany; 4 Smart-Q Software Systems GmbH, Lise-Meitner-Allee 4, 44801 Bochum, Germany; zenz@smart-q.de; 5 Paediatric Palliative Care Centre, Children’s and Adolescents’ Hospital, 45711 Datteln, Germany

**Keywords:** paediatric palliative care, participatory design, electronic health records, electronic medical records, technology acceptance, usability, user involvement

## Abstract

Background: Electronic medical records (EMRs) play a key role in improving documentation and quality of care in paediatric palliative care (PPC). Inadequate EMR design can cause incorrect prescription and administration of medications. Due to the fact of complex diseases and the resulting high level of medical complexity, patients in PPC are vulnerable to medication errors. Consequently, involving users in the development process is important. Therefore, the aim of this study was to evaluate the acceptance of a medication module from the perspective of potential users in PPC and to involve them in the development process. Methods: A qualitative observational study was conducted with 10 nurses and four physicians using a concurrent think-aloud protocol and semi-structured qualitative interviews. A qualitative content analysis was applied based on a unified theory of acceptance and use of technology. Results: Requirements from the user’s perspective could be identified as possible influences on acceptance and actual use. Requirements were grouped into the categories “performance expectancies” and “effort expectancies”. Conclusions: The results serve as a basis for further development. Attention should be given to the reduction of display fragmentation, as it decreases cognitive load. Further approaches to evaluation should be taken.

## 1. Introduction

Paediatric palliative care (PPC) is an approach to the care of children, adolescents and young adults with life-limiting and life-threatening illnesses [1]. The holistic and multidisciplinary approach addresses patients’ quality of life and the support of their relatives [2]. Globally, it is estimated that approximately 21 million persons require PPC [3]. Complex diseases, such as neurological, genetic and congenital diseases [4,5] or metabolic syndromes [6], are often rare [7]. In this regard, oncological diseases account for a smaller proportion of complex diseases [8]. One area in which PPC focuses is the management and alleviation of symptoms such as pain [9], anxiety and restlessness, [10] and sleep disturbances [11].

Due to the prolonged and complex disease trajectory, the information requirements for treatment and coordination of care are high [12]. In this context, electronic medical records (EMRs) play a central role in improving documentation and coordination in and quality of medical care [13]. Furthermore, EMRs can improve work efficiency and communication among health care professionals and increase accessibility to medical information [14].

However, the electronic medication process used by EMRs in PPC is challenging. There is a high probability of polypharmacy [15], and the use of off-label or unlicensed medications is common [16]. Due to the medical complexity and physiological characteristics of patients in PPC, they are particularly vulnerable to medication errors [17].

In addition to the advantages, however, there may be unintended consequences associated with the implementation and use of EMRs [18,19,20]. For example, there is a relationship between poor usability of EMRs and rates of burnout among nurses and physicians [21,22]. Poor usability of EMRs is also associated with negative well-being [23], cognitive failures and stress [24], (alert) fatigue [25], and high cognitive workload [26]. In addition, poor usability can potentially jeopardise patient safety, for example, by influencing the likelihood of medication errors [27]. The implementation of EMRs leads to changes in the work processes of nurses and physicians, which negatively affect their work habits [28]. A main reason for unintended consequences and dissatisfaction by health care professionals is the lack of user involvement in the development of EMRs [29].

Since inadequate EMR design can be a cause of the incorrect prescription and administration of medications, a careful evaluation of their usability is necessary [17]. Moreover, the unique needs of paediatric patients must be addressed in the context of EMRs and the electronic prescribing and documentation of drugs [30].

Therefore, EMRs must be developed to meet the needs of users and the specific requirements of the intended setting [19]. This point applies to general paediatrics and, especially, to the setting of PPCs as a result of the unique documentation needs of these cases [31,32].

A common approach used to involve (future) users in the development of new technologies is participatory design [33,34]. Participatory design is defined as a creative development process with four guiding principles [33]: (1) democratisation of decision-making processes; (2) mutual learning; (3) observation of latent (implicit) knowledge and (4) mutual creativity through collaborative work among developers, researchers and (future) users. To ensure user acceptance in the process of participatory design, theories such as the “unified theory of acceptance and use of technology” (UTAUT) prove to be the theoretical foundation [35,36]. UTAUT incorporates four core direct determinants of behavioural intention to use a technology from a user perspective: performance expectancy, effort expectancy, social influence and facilitating conditions. For this reason, UTAUT provides a useful framework for evaluating EMRs [37,38].

Insufficiently, many EMRs have been specifically developed for PPC in Germany. Therefore, an EMR for PPC was developed in the ELSA-PP project by adapting an EMR from adult palliative care to the context of PPC. Based on focus group interviews and one-on-one interviews with health professionals working in PPC, requirements were obtained [32]. Thereafter, a new patient chart module was developed and evaluated with the assistance of health professionals working in PPC [39]. In this study, one novel medication module was evaluated with the assistance of health professionals.

The aim of this study was to evaluate the acceptance of the medication module from the perspective of potential users in PPC and to involve them in the development process.

Research question: How do potential future users in PPC perceive the medication module in terms of technology acceptance with a focus on performance expectancy and effort expectancy, and what are their desires for improvement?

## 2. Materials and Methods

### 2.1. Study Design

A qualitative observational study was conducted in February and March 2021. For this purpose, the concurrent think-aloud (CTA) method was applied, followed by semi-structured qualitative interviews. Both methods were established by means of involving potential users in the development of new technologies [40].

When engaging users in the development of technologies, CTA is considered to be a “gold standard” [14]. During CTA, participants are asked to “think aloud” by verbalising their impressions and thoughts about a user interface during the use of software [41,42]. Data on observations and the self-reported statements of users regarding human–computer interactions can be obtained simultaneously [43]. Through CTA, subjective emotional and cognitive processes can be ascertained through verbal expressions, which are otherwise considered to be a “black box” of human thinking.

The ethics committee of the Witten/Herdecke University provided ethical approval for this study (approval code: 35/2019).

### 2.2. Participants

PPC professionals working in a PPC unit of a children’s and adolescents’ hospital in Germany were asked to participate in this study. Recruitment posters and leaflets were distributed to the PPC unit to inform participants about the study’s aim and procedure. Most PPC professionals were familiar with the procedure from a previous study [39]. After expression of interest in participation, materials concerning informed consent and study information were provided. Eligible participants included nurses or physicians who: (1) were actively working in PPC in the unit and (2) provided informed consent. No specific competencies or knowledge concerning the application of EMRs were required.

For participation, an expense allowance of EUR 40 per hour was offered.

### 2.3. Medication Module of the EMR

The medication module contained two key elements: (1) a view for the prescription of medications and (2) a view for the confirmation of medication administration. Both modules were developed as part of the ELSA-PP project and integrated into the software ISPC (company: smart-Q, Germany), which is used for adult palliative care.

**Figure 1:**
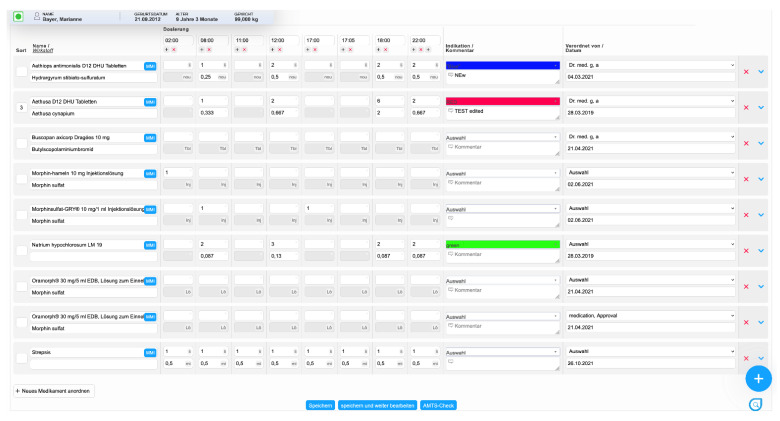
View for the prescription of medications (ISPC/smart-Q).

**Figure 2:**
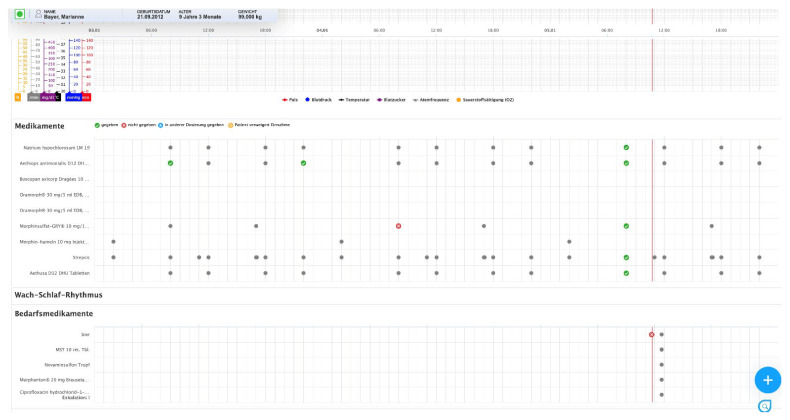
View for the documentation of medications in the patient chart module (ISPC/smart-Q).

(1) In the first view, physicians can prescribe medications and provide additional information about administration via comments (Figure 1). After the prescription, the medications are transferred to a patient chart module for documentation in clinical practice [39]. When prescribing, medications can be selected from an index (MMI Pharmindex) and the time, indication, interval, and comments for the prescription can be specified;
(2) In the second view, prescribed medications are displayed, and their administration can be documented (Figure 2). This view includes other parameters, e.g., concerning whether a medication was not administered to the patient as prescribed (different dose, different time) or whether a medication was not given/rejected (e.g., due to the fact of vomiting). This view is integrated with a patient chart module [39]. In the patient chart module, different parameters, such as vital signs or symptoms, can also be documented.

**Figure 3:**
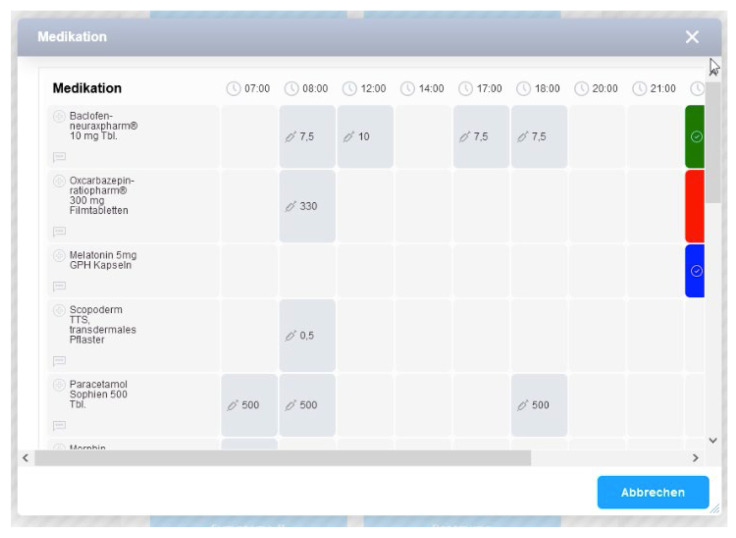
Plus-button menu in the patient chart module (ISPC/smart-Q).

The administration of drugs can be documented in two ways: (a) directly in the patient chart module by clicking on the grey dots or (b) via a central documentation plus-button menu (Figure 3). The plus-button menu is always visible in the EMR. This function permits quick entry of medication administration or other information while in other areas of the EMR (e.g., when performing care planning). This additional option is intended to account for the fast pace of work in the PPC unit.

### 2.4. Data Collection and Procedure

In addition to the medication module, other modules with the same procedure were also tested in the underlying CTA.

At the beginning of a session, participants were informed about the methodical procedure. According to their clinical role, different tasks were handed out on a printed sheet to structure the testing sessions (Appendix A). The tasks represented typical activities that nurses and physicians have to perform in clinical practice. Via a short and goal-oriented phrase, tasks specified what actions had to be performed, for example, “prescribe drug -a- at time -b-” [44].

Participants were encouraged to read each task aloud and to verbalise their thoughts continuously. If they forgot to perform these actions, they were reminded and encouraged by the interviewers. A decrease in or omission of verbalisation is a known behaviour of study participants using CTA, as verbalising their own thoughts is perceived as unfamiliar or uncomfortable [45]. Moreover, participants were told that there was no “right or wrong” action in the execution of the tasks and that the aim of the research was not to “test” the participants. Only a brief introduction to the application of the medication module was provided to evaluate the self-explanatory nature and learnability of the module. In addition, participants were encouraged to express desires and suggestions for further improvement according to their needs. If participants encountered problems with the application, sample questions were asked to help them explain the problem in greater detail (Appendix A).

After testing, a qualitative interview based on a semi-structured guideline (Appendix B) was conducted. The interview contained questions about the participant’s overall impression of the medication module and its usability and content as well as suggestions for improvement from the perspective of participants. A demographic questionnaire was administered following the interview.

Two researchers with experience in CTA were present at all testing sessions. A hardware setup consisting of a desktop computer, a screen for participants and a mirrored screen for observation from a regular office was installed. Screen movements and audio were recorded as a screencast with Captura (version 8.0).

### 2.5. Data Analysis

Audio files were transcribed verbatim following the transcription rules of Dresing and Pehl [46]. Then, structuring qualitative content analysis was applied to the transcripts and videos with MAXQDA (2020) using inductive and deductive methods [47]. The determinants of UTAUT served as the theoretical basis for the analysis. First, observations and statements were deductively assigned to the UTAUT determinants performance expectancy and effort expectancy (Table 1). Inductive subcategories were formed within the determinants. These determinants are therefore highly relevant because they focus on the software itself. Focus on these determinants allows the content of the software to be concretely improved.

The analysis was performed independently by two researchers (S.K., C.J.). The categories and subcategories were discussed until a common consensus for coding was reached. Subsequently, codes were discussed among the research team until consensus was reached (T.S.B., L.A.D., D.M.).

For the presentation of the results using verbal quotations, pseudonyms were assigned (profession_01_#00:00:00#), and the quotations were translated into English.

## 3. Results

### 3.1. Participant Characteristics

Fourteen CTA sessions were conducted with 10 nurses and four physicians over a 4 week period in March 2021. Of these participants, 12 were female and two were male. Participant characteristics are shown in detail in Table 2. The mean duration of a session was 61 min, with a range of 45–99 min. The mean duration for testing the medication modules was 31 min with a range of 22–44 min.

### 3.2. Main Findings

**Figure 4:**
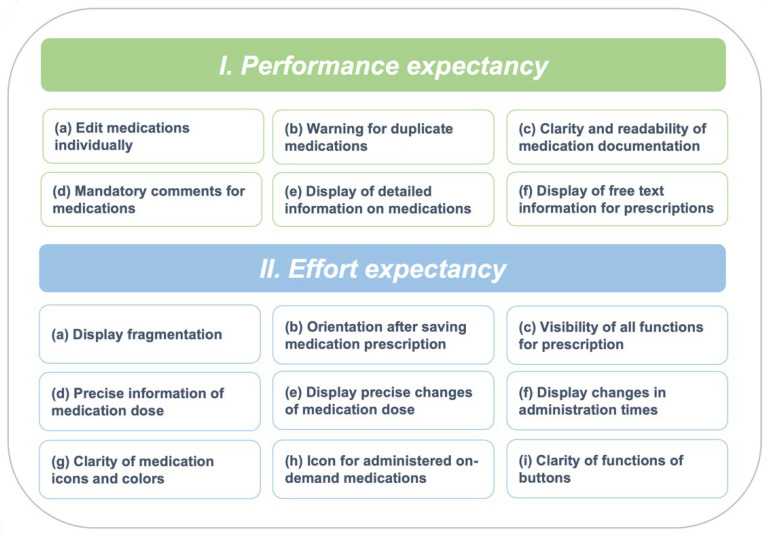
Categories and subcategories from the qualitative content analysis.

Deductively, two main categories were grouped: (1) performance expectancy and (2) effort expectancy (Figure 4). Within the main categories, inductive subcategories were grouped. In total, 649 subcodes were assigned (35 codes minimum–74 codes maximum per session). Because of the high number of observations and statements, only particularly serious or frequently named observations and contents are reported in this manuscript.

In the following, the results of the individual modules are shown together with the main categories. The module to which the results relate will be listed.

#### 3.2.1. Performance Expectancy

- **(a) Edit medications individually**

In the *view for the prescription of medications*, it was possible to click a button and then edit all medications as needed. After that, a button could be clicked to save all changes. It was suggested that one medication could be edited individually instead of editing all medications at the same time.

*I would like it if I had the possibility to select this [a single medication] directly somehow and then also to change only one single medication. So that’s [editing all medications] also great if I have a lot of medications that I want to change something, but if I only want to change one at the moment, but I have 25 medications there, then I first search.*

(Physician_07_#00: 35:25#)

- **(b)** **Warning for duplicate** **medications**

As a new feature in the *view for the prescription of medications*, it was proposed to display a warning sign when prescribing a medication that has already been prescribed.

*And the drug now appears three times, because apparently someone has already worked on it. Then I would like to have either, if this is to remain so, a warning notice that it is now listed a number of times.*

(Physician_07_#00: 44:20#)

- **(c) Clarity and readability of medication documentation**

The *view for the documentation of medications* was perceived as clear and readable. In particular, adding contextual information via a free text field before saving the documentation was considered useful.

*So, the presentation (of medications), when the administration times are, I think that’s good. Very clear. I think, I can also add times and so, if there are other times. That’s all possible. I found that quite good. The changes went well. [...]. Or, also, there are comments for when I did not give it. [...] I think that is also quite good. If the patient then refuses, it also happens sometimes. [...].*

(Nurse_05_#01:13:36#)

- **(d) Mandatory comments for medications**

Additionally, it was suggested that comments could be made mandatory when documenting medications in the *view for the documentation of medications* if deviations from the prescribed medication have to be documented. For example, if a medication could not be given to the patient for certain reasons, e.g., vomiting.

*Then, I like the fact that you can immediately see what medications I have documented as given. And that I can also go over it and see if there’s another comment. And yes, it should almost be something that you have to fill out, why you didn’t give something. You could still think about that. Because the order is made by the doctor, but the nursing staff are the ones who have to document that they have given medication and also when. And that should, could perhaps be discussed again, whether one does not have to comment on deviations from the medication plan in principle. That, otherwise, you practically can’t close the window.*

(Nurse_05_#00:46:15#)

- **(e) Display of detailed information on medications**

Participants emphasised that the direct display of detailed information on medications in the *view for the documentation of medications* was useful. For example, participants were able to see what type of medication tablets were prescribed.

*Otherwise, I think it’s great, I think it’s very clear that everything is on top of each other, yes. What I also find totally good, I just now see that it also says how much, that these are film-coated tablets, for example, or that this is a solution or suppositories, that you have this information somehow directly on top of each other. Because we often have medications where the manufacturer’s name changes again and then you stand there and think to yourself, great, is this juice or is this a tablet and then you start looking. [...]. I think that’s good, yes.*

(Nurse_09_#00:21:35#)

- **(f) Display of free text information for prescriptions**

In the *view for the documentation of medications,* it was found that the information entered for a particular prescription by the physician in a free text field was not transferred to the patient chart module. As a result, participants were irritated, as information on the preparation of the prescribed medication was missing.

*What do I see here now? Morphine is there. I don’t see it there then, do I? How that is manufactured. There I would have to then practically in the medicines. Exactly.*

#01:00:44#

*Yeah, at least with an icon or something. That you don’t, yeah, how do you do that? You know, there’s a hint there. Maybe a call sign or something. So that one knows, I have to go into the medication plan once, so I know how to set it up. I think that would be enough. Because then once you know it, then you know where I can look.*

(Nurse_05_#01:01:28#)

#### 3.2.2. Effort Expectancy

- **(a) Display fragmentation**

In the *view for the prescription of drugs* the participants perceived display fragmentation. With an increasing number of medications, the display where the time for the prescription could be entered moved out of the display window when scrolling down. As a result, participants had to scroll up and down to make sure they had entered the correct time. Participants felt it was important that the display where the time for the prescription could be seen was visible at all times during the process of prescription.

*At eight o’clock and five o’clock. So, if I already have times, I would find it cool if that would come along [the display], because now I don’t have to plan anymore for the time [for prescription], I’m actually there.*

(Physician_07_#00:39:48#)

- **(b) Orientation after saving prescribed medications**

After saving a prescription in the *view for the prescription of medications,* participants did not feel confident that changes had been saved. This lack of confidence stemmed from the fact that the entire view of the display shifted to the top of the screen after saving. After that, it was necessary to scroll down again to see the prescribed medications. Participants described that this shift made it difficult to reorient oneself due to the large number of medications.

*Now, I have to save it down here. I don’t know if that’s unusual, but I find it somehow not very* *intuitive. And now you end up somewhere completely different [after saving a prescription]. Now, you’ve saved, and now you have to go back and find where that medication is, if it’s anywhere at all. Am I too far? I put it at the end. Here, there it is.*

(Physician_03_#00:34:16#)

- **(c) Visibility of all prescription functions**

A frequently observed problem in the *view for the prescription of medications* was that participants did not see a submenu containing information concerning the calculation of the dose of a medication according to body weight. The submenu could be expanded by an arrow leading to a dropdown menu on the right side of the screen (Figure 1). Here, detailed information about the prescription could be entered, e.g., specific concentration, unit of dose, route of administration and dosage form.

*I: But it irritated you now that what you are supposed to do and where to find all the prescription functions was not immediately displayed. Is that right?*

*P: Yes, exactly. But this, I think is great, I have to say. After I had just said. I’m going to retract* *that. I didn’t know what was coming [in the submenu]. I think that’s totally good that it says, one tablet equals xy grams per body weight. And then so and so is so many. Right? That the calculation of the dose according to body weight is probably done automatically.*

(Physician_11_#00:30:45#)

- **(d) Precise information concerning medication dose**

In the *view for the documentation of medications*, participants expected the module to display precise information concerning a medication dose. In one case, participants were not clear about the specific dose to be administered. It was found that, for medication, in addition to the dose per milligram, the number of milligrams to be administered should also be indicated (e.g., rivotril at a dosage of 2.5 mg per ml, and so for 3 mL to administer 7.5 mg). This oversight resulted from the fact that in the MMI Pharmindex, in addition to the drug, the dosage per ml/tablet, etc., was also selected (e.g., selection of ibuprofen 600 mg or ibuprofen 400 mg).

*But that’s confusing, because if I now understand correctly, then it says with baclofen, for example, dosage 7.5 mg. So, it’s very clear to me, okay, I’m giving 7.5 mg. But with rivotril it says dosage 2.5 mg per millilitre, and there you could now also assume that that is the bottle indication and not what I should give.*

(Nurse_01_#00:16:59#)

- **(e) Display precise changes to medication dose**

In the *view for the documentation of medications,* participants expected the module to display precise changes to medication dose after documenting the administration if the administered dose differed from the prescribed dose. Participants appeared irritated that the number representing the dose (e.g., 10 milligrams) in the display did not change to the actual dose administered (e.g., 5 milligrams). Additionally, here, it was not perceived as sufficient to display the information concerning changes to medication dose in a mouse-over field or to display a specific blue icon as the dose was given.

*Now, it irritates me that there are still the five [milligrams], although I just entered 10 [milligrams]. [...]. I think that’s good that it’s in the overview, so now from that it’s immediately apparent that it was taken or it wasn’t taken [by the patient]. I would have wondered, that’s why I clicked it again, whether the blue [icon] now means it was taken or something else. It would irritate me now in the overview just that the designation has remained so. [...] I think it should also be in the overview that it was ten milligrams. Because this way I would assume that five milligrams were given if I give it now and not that this is the changed dosage.*

(Physician_07_#00: 31:11#)

- **(f) Display changes in administration times**

If the time of administration differed from the prescribed time, participants expected the module to display the changes in administration times of the regular medications in the *view for the documentation of medications*, for example, a case in which a medication was prescribed for 7 a.m. and for some reason (e.g., morning care) it could not be given to the patient until 9 a.m. Participants appeared irritated that the icon indicating the administration of a given medication did not change the position on the clock. Instead, the icon remained at the original time marker where the medication should have been given (i.e., 7 a.m.). It was not perceived as sufficient to display information concerning changes in administration times in a mouse-over field as already available in the module.

*It doesn’t do that now with the time [of administration], for example. I would still think that it might be quite practical if it [the icon] were to slide over here, so that you could see directly, okay, he didn’t get it [the medication] at seven, but at nine. Because I wouldn’t look at this button [the mouse-over field] again for every patient who gets 15 medications in order to look at it, exactly. I would find that quite good, if that is then perhaps also somehow marked with such an icon, so that you can then just go to it and then see the comment, I don’t know, did the patient got that medication somehow because of the morning care then only later or has the patient vomited?*

(Nurse_09_#00:15:13#)

- **(g) Clarity of medication icons and colours**

A feeling of ambiguity was expressed regarding the clarity of medication icons and colours and the representation of meaning for regular medications in the *view for the documentation of medications*. Most participants were able to interpret the meaning only after applying the functions. However, it was mentioned that the meaning of the individual symbols becomes comprehensible after a period of use.

*And everything [the symbols] has a different colour. That I see directly: Something was going on. And if we have the colours on it, we also know directly what it was, probably. [...]. So, if a check mark is clear, then it’s [the medication] given. [...] With the red cross: not given. Now what is the asterisk icon? I would not have guessed what that means. But that I gave a different dosage, I don’t know how else to show that, that you know that I gave something else or less or more. And just like here with the yellow icon. But if it’s always the same icon, then it’s obvious at some point. So now we also have some symbols that we enter in the curves.*

(Nurse_06_#00:18:22#)

- **(h) Icon for administered on-demand medications**

When documenting on-demand medications in the *view for the documentation of medications*, the icon for administered on-demand medications did not change the symbol. For regular medications, it was assumed that the icon would change to a grey colour and symbol after saving that a medication was given.

*Ah, yes, I click on it [the icon for the medication]. Did I give it an hour back? Yes, then. 1:50 p.m. Save. [...] So, what does it [the software] do with the medication? It loads and it [the icon] stays grey. Oh, okay. But theoretically it [the icon] would have been there now as a green check mark.*

(Nurse_06_#00:22:07-5#)

- **(i) Clarity of button functions**

In the plus-button menu of the *view for the documentation of medications*, the functions of different buttons were not perceived clearly. For example, the function of a button for documenting multiple medications [e.g., 8:00 a.m.] was not discovered by most participants. This oversight could be attributed to the fact that the button was not perceived to be clickable.

*That was my first thought, I click on it like this and then…Because for me the [icons with] clocks are somehow like this, they are just not displayed so prominently. But rather in the grey tone, which is rather unnoticeable. For me, this is rather always a sign that the button is not selectable. I say times. That’s why I didn’t even think about the fact that you could also click on the time.*

(Nurse_01_#00:19:35-3#)

## 4. Discussion

The aim of this study was to evaluate the acceptance of a medication module in terms of performance expectancy and effort expectancy from the perspective of potential users in PPC and to involve them in the development process.

Requirements from the user’s perspective could be identified as influences on the acceptance and actual use of the medication documentation module. These requirements in the areas of performance expectancy and effort expectancy provide a basis for the further development of the modules. The nurses and physicians requested and discussed similar aspects for the improvement of the software, especially when considering the effort expectancies. Nevertheless, all participants could also describe their personal wishes and important and unique feedback could be collected.

In the category of performance expectancy, several important user requests were identified. For example, users requested a display of warnings concerning inappropriate drug duplication. A systematic review of factors contributing to medication errors when using computerised order entry in paediatrics has shown five major factors: (1) lack of drug dosing alerts; (2) generation of inappropriate dosing alerts; (3) inappropriate drug duplication alerts; (4) dropdown menu selection errors, and (5) system design [17]. Therefore, integrating a function for drug duplication alerts should be a potential aspect for further development of the modules with the aim of avoiding medical errors.

Moreover, displaying a free text field in the view for the documentation of medications in the patient chart module is also an important feature to implement. Since it is common in paediatrics to use off-label medications [16] or nonstandard doses or volumes, paediatricians are often forced to write specific instructions for preparing medications or other information in a free text field [17].

Additional attention should be given to requirements in the category of effort expectancies because that category encompasses usability and complexity of use. It is necessary to identify aspects in this category and to adapt the module accordingly because poor usability can contribute to patient harm [48]. For instance, Ratwani et al. found that failures to provide appropriate feedback, such as confusing or cluttered EMR information, can contribute to patient harm in paediatric settings [27].

Particular attention in the further development of the modules should be given to the need for unambiguity and precision concerning doses. A scoping review by Conn et al. found that dosing errors or medication formulations are central factors for the occurrence of medication action errors in the paediatric population [49]. Therefore, it is necessary that the dose of a drug is both precisely prescribed in the view for the prescription of medications and transferred without the possibility of misinterpretation of the dose to the view for the documentation of medications. For example, specific explanations of medication dosages should be added in a free text field when prescribing to avoid misinterpretation [30]. Special attention must be given to this problem in further development.

Furthermore, a display fragmentation was identified, particularly in the module for prescribing medications. This fragmentation occurred especially when medication lists were particularly long, which is often the case in PPC [15]. Display fragmentation is a particular problem in the application of EMRs, as they negatively affect the cognitive load of users. This fragmentation requires users to repeatedly scroll back and forth between information and to store information in their working memory [50]. This necessity can influence stress for users in addition to the already existing stress factors of clinical practice [26].

Another factor that requires attention in further development is the reduction of irritating data entry requirements, which could be identified in the module for prescribing medications. Data entry should be particularly clear and intuitive for users to avoid confusion and possible errors in data entry. In a study of paediatric settings, it was found that confusing data entry is a relevant usability issue [27].

In addition to the factors identified here that influence user acceptance, there are other factors to be considered that influence the quality of the application. For example, sufficient training of users is necessary, which can increase the quality of documentation [51]. Insufficient training in the use of EMRs is considered to be a major barrier to their adoption [14] and poor documentation can potentially lead to medical errors [51]. Furthermore, as a principal approach to the safety of patients, the American Academy of Pediatrics suggested three components in a policy statement: “(1) awareness of the epidemiology of errors and the institution of methods for error identification; (2) the integration of improvement science, including a safety culture, into daily work; and (3) the creation and implementation of core patient-safety solutions.” [52]. Despite the potential of implementing and using EMRs, measures beyond the technology, per se, are necessary to improve safety and the quality of documentation.

As outlined in this study, transparent evaluation with the participation of future users of EMRs is highly relevant. As discussed in Section 1, it is important to involve potential future users in the development process as early as possible to ensure that EMRs are meeting their needs and the requirements of the context of the PPC. If the vendors of EMRs that develop and distribute such software adapt the usability without involving the users, there is no guarantee that the needs will be addressed.

## 5. Limitations

The results must be considered against the backdrop of the methodological approach and study conditions.

Potentially, the results are influenced by the presence of two observers using a CTA approach. It is known that participants may adjust their behaviour and statements in a positive direction in such situations due to the fact of social influence [53]. In addition, the natural thinking process of participants may have been influenced by the need to think aloud [41].

Furthermore, the results were obtained under laboratory conditions. For example, the application of EMRs often takes place under time pressure and conditions of psychosocial stress [24,54]. In general, EMRs are complex systems with multiple views and functions [14,55]. As a result, future approaches should also evaluate the interaction of the medication module with other views and functions of the EMR. It can be assumed that the results would be different under real clinical conditions.

In addition, it must be noted that the development of digital technologies and, in particular, the testing of their usability takes place in iterative steps. After redesign of the module, it is recommended that further forms of evaluation be carried out. Here, additional approaches to evaluation with users should be considered. More formalised, user-oriented approaches could be applied. As Schaaf et al. suggested, “near live clinical simulations” could be useful [56]. In such approaches, scenarios are constructed to reproduce the clinical environment to simulate a more realistic testing situation [56]. Under such conditions, it is also possible to simulate workflow interruptions that often occur in clinical practice [44]. Approaches to CTA and “near live” approaches could be seen as complementary methods. However, “near live” approaches address workflow issues and provide more realistic data under simulated clinical conditions [53]. Moreover, it is recommended that after further iterations with users, expert-based approaches to evaluation be adopted such as cognitive walkthroughs [50]. In an expert-based approach, the software is evaluated by usability experts with predefined tasks.

A further limitation is related to the size of the sample. There is much debate in the literature on evaluating the usability of digital technologies regarding the necessary sample size. Regarding the specific evaluation of usability, it is assumed that 80% of all usability problems can be identified by five users [57]. Other authors argue that more than 10 participants are necessary to identify all major usability issues [41]. In principle, it is recommended to conduct several iterative cycles with fewer participants rather than to test many users directly [41]. For the user group of nurses, a sufficient number of participants could be reached for this study. Sufficient data saturation was observed in the evaluation process. In contrast, only four users could be recruited for the user group of physicians. This fact can be seen as a central limitation, especially for the module on the prescription of medications. However, it must be noted that only approximately 10 physicians worked in the PPC unit involved in the study. Although physicians described similar aspects in terms of effort expectancies, it cannot be ruled out that more participants would identify additional aspects. In additional interactions, a further attempt should therefore be made to recruit a higher number of physicians.

However, against the backdrop of the results, we are confident that the findings are rich in content and well-suited to adapt the tested modules to perform further cycles of evaluation. In further steps of evaluation, it is important to recruit a larger number of physicians.

## 6. Conclusions

This study identified key factors that serve as potential influences on user acceptance in the context of the UTAUT dimensions of performance expectancy and effort expectancy. The results serve as a basis for the further development of the modules evaluated here, which must subsequently be implemented. Particular attention should be given to the reduction of display fragmentation, as it reduces the cognitive load of the users. Since PPC patients are particularly susceptible to medical errors, further approaches to evaluation should be taken.

## Figures and Tables

**Table 1:** Definitions of the core direct determinants of UTAUT [36].

UTAUT Determinant	Definition
Performance Expectancy	“The degree to which an individual believes that using the system will help him or her to attain gains in job performance”, which encompasses mainly the functions of a technology.
Effort Expectancy	“The degree of ease associated with the use of the system”, which basically encompasses the dimensions of perceived usability and complexity of use.

**Table 2:** Overview of the participants’ characteristics.

Characteristics	
Sex	
Female	12
Male	2
Age in years (mean) ^1^	43
Profession ^1^	
Physician	5
Nurse	9
Years of PPC experience ^1^	
0–9	3
10–20	5
>20	5
Years of experience in current position (PPC) ^1^	
0–9	6
10–20	5
>20	2
Participants with experience in professional use of EMR ^1^	3
Experience in professional use of EMR in years ^1^	
0	11
1–4	1
5–8	0
≥9	1

^1^ Characteristics from one participant are missing due to the nonreturn of the questionnaire.

## Data Availability

The data presented in this study are available upon request from the corresponding author.

## Appendix A

### Appendix A.1. Tasks for Nurses

- Document a drug in the morning medication routine as given in the patient chart module; view the documentation in the patient chart module;
- Document that a drug in the morning medication routine was given at a different time than scheduled;
- Document any medication as given; change dosage when given;
- Document any medication as not given and comment as desired;
- Document that any medication was refused;
- Switch to the plus button menu;
- Document all drugs for any time as given (e.g., all drugs at 08:00);
- View the documentation in the patient chart module;
- Document in the patient chart module that you gave any on-demand medication one hour ago.

### Appendix A.2. Tasks for Physicians

- Document that any medication was given at a different time at double the dose;
- Document any drug as not given and comment as desired;
- View all documentation in the curve view;
- Click on the “Medication” button in the blue menu bar at the top and get an overview;
- Prescribe the following medication:
- Topiramate–1 A Pharma^®^ 25 mg Film-Coated Tablets^®^: 25 mg 08:00 and 100 mg 17:00. The medication should only be scheduled for 7 days, starting tomorrow;
- On demand medication:
- BUCCOLAM^®^ 5 mg solution for oral use for seizure >3 min. o. series, escalation level I.

### Appendix A.3. Sample Questions for Problems

- Describe the problem you are having right now?
- What would you expect to happen here?
- How would you solve the problem?
- What would you like to see happen?

## Appendix B. Interview Guide

- What were your first thoughts when you applied the new content (of the medication module)?
- How did you feel about the application? What did you like about the application? What did you not like about the application?
- Do you think the new content helps you to get a better overview of the patients?
- Medication is an area in which patient safety plays a particular role: Do you think the new content has an impact on patient safety? If yes, to what extent?
- Depending on your answer, what would need to be adjusted to improve patient safety in your opinion?
- Are there any challenges that might arise in dealing with the new content?
- Do you have any additional desires after using the new content? As an addendum, if applicable, are there things you would like to add?
- How do you think your colleagues would evaluate the new content?
- What do you think other colleagues would rate as positive?
- A final question concerning this testing situation, (add if necessary) did you find it easy to verbalise your thoughts?
- What made verbalisation more difficult for you?
- What would make it easier for you to verbalise?
